# Cognitive impairment in individuals with low educational level and homogeneous sociocultural background

**DOI:** 10.1590/S1980-57642014DN84000007

**Published:** 2014

**Authors:** Sonia Maria Dozzi Brucki, Ricardo Nitrini

**Affiliations:** 1 MD, PhD. Cognitive and Behavioral Neurology Group - University of São Paulo, São Paulo SP, Brazil.

**Keywords:** cognitive impairment, dementia, illiteracy, cognitive evaluation

## Abstract

Prevalence studies of dementia and cognitive impairment in different cultures and environments are necessary.

**Objective:**

To verify the prevalence of cognitive impairment and dementia in a rural homogeneous population from flooded areas in the Amazonian Basin.

**Methods:**

A total of 163 subjects were interviewed with neurological, cognitive and functional evaluation. The individuals were classified as demented or cognitive impairment no dementia (CIND).

**Results:**

cognitive impairment was observed in 11.4% (n=18 individuals). Ten out of the 18 were diagnosed as CIND and eight with dementia. The prevalence rate of dementia was 4.9% in subjects aged 50 years or over and of CIND was 6.1%. Considering only the elder group (>64 years of age), there was a 12.3% prevalence of dementia and 7.7% of CIND.

**Conclusion:**

In a homogeneous population, we observed a similar prevalence of dementia to rates reported by studies in Brazil and in other developed and developing countries.

## Abstract (pt)

Estudos de prevalência de demência e de comprometimento cognitivo em diferentes culturas e ambientes são necessários.

**Objetivo:**

Verificar a prevalência de comprometimento cognitivo e demência numa população rural homogênea de áreas alagadas da bacia Amazônica.

**Métodos:**

Entrevistamos 163 indivíduos e avaliação neurológica, cognitiva e funcional. Foram classificados em dementados e comprometimento cognitivo sem demência (CCSD).

**Resultados:**

Comprometimento cognitivo foi observado em 11,4% (n=18 indivíduos). Dez deles foram diagnosticados como CCSD e oito com demência. A taxa de prevalência foi de 4,9% em sujeitos com idade igual ou superior a 50 anos e 6,1% de CCSD. Considerando-se somente os idosos (>64 anos) a prevalência foi de 12,3% para demência e 7,7% para CCSD.

**Conclusão:**

nesta população homogênea nós observamos uma prevalência de demência similar a outros estudos em nosso país e em outros desenvolvidos e em desenvolvimento.

## INTRODUCTION

Dementia is very common in the elderly population Prevalence of the disease doubles every five years in individuals over 65 years of age.^1^ Epidemiological studies from many countries have demonstrated different prevalence rates of dementia. This heterogeneity could be explained by differences in age, instruments used for cognitive and functional evaluations, diagnostic criteria (DSM-III, DSM-IV, DSM-IVR), rural or urban areas, and representational role of samples studied. Cognitive and functional evaluation instruments must be adapted for the environment in which they will be applied.

Ferri et al. have asserted that dementia prevalence varies widely around the world, ranging from 1.6% in Africa to 6.4% in North America among subjects aged 60 years and older.^2^ A review of community-based studies has demonstrated different prevalence rates. Prevalence in European studies range from 1.1% to 13.9%; in North America from 4.2% to 9.6%; in Africa, Asia and Australia from 1.4% to 22.1%; and in Latin America from 1.3 to 19%. Among Brazilian studies the prevalence of dementia is from 5.1% to 19%.^3^

Dementia prevalence is higher among older individuals, ranging from 1.2% among 65-69 year-olds to 54.8% among elderly over 95 years of age.^4^ Prevalence among the oldest old is much higher. In the 90+ Study (USA), the prevalence was 45% and 28% among women and men, respectively.^5^ Based on preliminary data from Sweden, in Göteborg, the prevalence reported among elderly over 95 years of age (approximately 950 subjects) was 56% in women and 37% in men, rising to 60% and 40% in women and men over 99 years of age.^6^

Some studies have observed a higher prevalence among individuals living in rural areas.^7-9^ A study with pooled-data from epidemiological studies in Latin America observed a higher prevalence rate of dementia among illiterates (15.7%) but a higher prevalence in younger ages compared to studies performed in developed countries. General prevalence in this study was similar to the rate observed in high-income countries.^10^

Prevalence of dementia decreases with increasing educational level, as observed in a recent systematic review which showed a dementia risk of 2.61 among subjects with low schooling (95% CI 2.06-3.33) compared to subjects with higher educational level.^11^

Risk factors associated with higher prevalence of dementia among illiterates and individuals with low educational level are: low cognitive reserve, poor control of cerebrovascular risk factors, difficulties during cognitive evaluation, and poor cognitive test adaptations in these populations. Besides lack of formal education, other bias factors are associated with income and socioeconomic aspects, such as childhood development and life expectancy, which could affect survival until more advanced ages.

In studies conducted with samples from large cities, subjects differ greatly, encompassing people from rural areas with distinct cultural backgrounds, different educational systems and professional activities with different levels of demand.^12^ Evaluation of subjects with analogous backgrounds and similar socioeconomic, cultural and environmental factors, allows observation of the prevalence of cognitive impairment besides the effect of schooling, yet such studies are rarely conducted. Therefore, the aims of this study were to determine the prevalence of cognitive impairment and dementia in a rural homogeneous population from flooded areas in the Amazonian Basin. These communities remain largely unknown, particularly in terms of the characterization of cognitive impairment and dementia.

## METHODS

**Figure 1:**
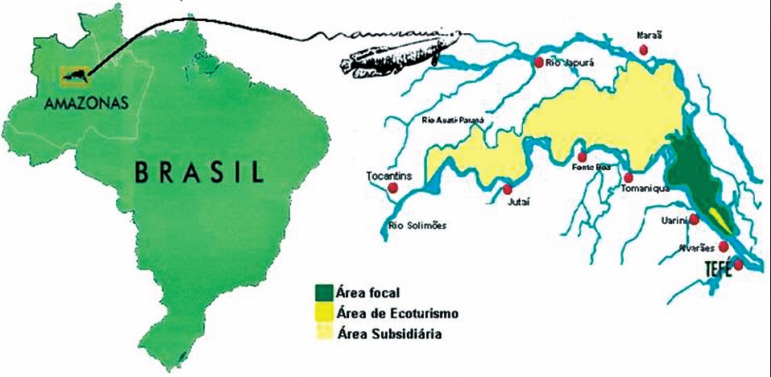
Mamirauá area.

**Figure 2:**
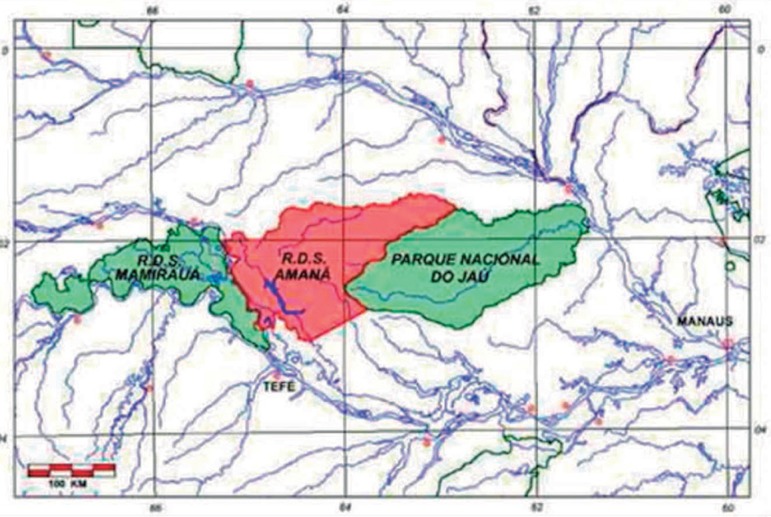
t

**Environmental setting and subjects**. The Mamirauá and Amanã Sustainable Development Reserves (MSDR and ASDR, respectively) are located about 600 km West of Manaus (Amazonas) in the Brazilian Amazonian region (Figures 1 and 2). The reserves are contiguous conservation units designed to integrate the preservation of habitats with sustainable development of local resident communities. These areas are seasonally flooded, with water levels rising 10 to 12 meters above normal in the rainy season. The population lives in small communities along the riverbanks, each comprising 13 domestic households on average, which are typically linked by kinship ties that characterize the communities as nuclei of small groups of related individuals. The houses are timber-built and raised off the ground to protect them from high water levels. It is essentially a subsistence-based economy, with very low incomes (annual family income of about US$900). Activities are divided among fishing, growing manioc for flour and in some communities, hunting; which is practiced throughout the lifetimes of most subjects. Only among the elderly do physical activities decrease. Women tend to work making flour, housekeeping and taking care of children. Almost all communities have schools that provide education for up to four years. The communities also have limited access to radio and are even more restricted in terms of TV, newspapers and telephone use. Electricity, when available, is produced by diesel generator and limited to a few hours per week. The distance of communities to nearest towns varies between nine and 18 hours by boat. The MSDR has 5,615 inhabitants, 435 of whom are aged 50 or older (7.7%), whereas the ASDR has 1,881 subjects, and 151 (8%) in the 50 or older age group (data provided by the Mamirauá Institute, census of 2002). There are 62 communities within the MSDR and 23 within the ASDR.

We carried out seven expeditions to the area, staying for 12 to 15 days on each visit. The visits to the communities were made by boat, enabling us to interview 43 different communities (23 in MSDR and 20 in ASDR), which were relatively close together (clusters of communities). Each community was visited only once, and all subjects aged 50 years or older who were present at the time of our visit were given a thorough general examination. We were able to evaluate 69.4% and 45.2% of the Mamirauá and Amanã populations older than 50 years, respectively. The sample population did not differ for age (χ^2^=0.043, p=0.836) or gender (χ^2^=1.53, p=0.216) compared to the total population of over 50s. Subjects were interviewed at their homes or in community centers where clinical, neurological, neuropsychological and anthropometric examinations were carried out, and blood samples collected for glucose and cholesterol level measurements.

Individuals were considered illiterate when they fulfilled all of the following three conditions: they had never attended school, or had attended for less than one year; they considered themselves unable to read, and were unable to read the phrase "close your eyes" from the Mini-Mental State Examination. The sample was divided into two age groups: 50-64 years (A - Adults) and >65 years (E - Elderly); and also by education: Group 0 - illiterates; Group 1 >0 years of schooling. None of the subjects were taking medications with central nervous system action.

**Subjective memory impairment**. In an interview with the subject, the answer to the question: "Do you have problems with your memory?" was used to classify subjects into groups with or without SMI.

**Neuropsychological evaluation**. The subjects were submitted to a range of tests adapted for the local situation. Memory, executive functions, temporal estimation, cancellation tasks, and verbal fluency were tested. Several tests were chosen for analysis in this study as follows:

Mini-Mental State Examination (MMSE):^13^ a version recommended for use in Brazil (Brucki et al., 2003). Adaptations were made for evaluating spatial orientation - name of community, nearest community, town and the nearest town.

Brief Cognitive Screening Battery (BBRC):^14^ consists of 10 line-drawings (shoe, house, comb, key, airplane, turtle, book, spoon, tree, and bucket) presented three times to the subject, and after about a five-minute delay the subject was requested to recall as many drawings as he or she could (delayed recall).

Verbal fluency Tests:^15^ semantic category - generation of animals in 60 seconds.

**Screening mood and mental symptoms**. Happiness Analogical Scale:^16^ A sheet of paper with seven line-drawings of circles representing faces showing varying degrees of happiness and sadness, with the fourth representing a neutral face, was presented to the subject. Subjects were asked to choose from among faces that best identified their own mood. Faces were scored as follow:

1 - very happy;
2 - happy;
3 - a little happy;
4 - neutral;
5 - a little sad;
6 - sad;
7 - very sad.

The scale is easy to explain and has few instructions, making it more straight forward for illiterates. For analyses, scores 1 and 2 were taken together as indicative of self-perception of happiness, while scores 6 and 7 were presumed to mean the opposite.

Patient Questionnaire (PQ) of the PRIME-MD, *Primary Care Evaluation of Mental Disorders.*^17^ This instrument was developed for diagnosing mental disorders by clinicians in primary care, with simple, easy-to-understand questions. The PQ serves as an initial symptom screen for mental disorders. We opted for the sum of items (ranging from 0 to 26) and read out the questionnaire to all interviewees, so as to minimize the effect of illiteracy. The questionnaire comprises 26 questions covering somatic symptoms (15); depressive symptoms (2); anxiety symptoms (3); eating disorders (1); and alcohol abuse (4) with higher scores indicating pathologic states. On completion, subjects classify their self-perception of health status as: poor, fair, good, very good or excellent (27^th^ item).

**Functional evaluation**. A subjective evaluation was used, considering activities carried out in the community and expected functionality by their peers.

**Criteria for determination of cognitive impairment and dementia**. First, subjects with cognitive impairment were classified: MMSE scores less than or equal to 14 for illiterates and less than or equal to 19 for literates (means - 2 SD)^13^ and lower-than-recommended scores for the animal fluency test and/or delayed recall on the BCSB,^15,18^ analysis case by case observing other tests, and final clinic impression. Memory complaints were also considered. Dementia diagnosis was based on DSM-IV criteria and functional decline in activities of daily living, whereas cognitive impairment no dementia was considered when there were low scores on cognitive tests (previously described) and preserved functional activities.

**Statistical analyses**. Analyses were performed with Biostat 4.0 or Statistica 10.0 software. Nonparametric tests were used (Mann-Whitney Test) for continuous variables and independent variables and Chi-square tests for ordinal variables The study was approved by the Research Ethics Committee of the Hospital das Clínicas of the São Paulo University School of Medicine and of the Mamirauá Institute. All subjects gave written consent for their participation in the study, or relatives had given written consent on behalf of those subjects unable to sign.

## RESULTS

**Demographic aspects**. We evaluated 163 participants with a mean age of 62.3 years (±9.16 y), ranging from 50 to 94 years, and a median value of 60 years. Gender distribution was evenly balanced (82 females and 81 males). Three women were excluded because of blindness (1), deafness (1) and aphasia (1). Individuals included in the sample were in good general health, having a very low prevalence of arterial hypertension and of vascular risk factors. There were no cases of dyslipidemia and three of arterial hypertension.

This sample was characterized by very low educational level, with median value of zero, a mean of 0.83 y (±1.55y) and range from 0 to 11 years. The distribution by schooling was: Group 0 (illiterates)=110 subjects (67.5%); Group 1 (>0 y)=53 (32.5%), with only four individuals with more than four years of schooling.

The age groups were distributed as follows: the adult group (50-64 y) had 98 subjects (60.1% of sample), a mean age of 56.1 (±4.1) years and median age of 56 years, whilst the elderly group (>65 y) comprised 65 subjects, with a mean age of 71.8 (±6.2) and median age of 70 years. There was no difference in the proportion of gender in these groups (χ^2^=0.30; p=0.586). Furthermore, 159 subjects (97.5% of sample) were considered functional illiterates. There was a significant difference in educational level across age groups (p<0.001) and between genders.

Diagnosis of cognitive impairment was based on data from cognitive and functional evaluations. Cognitive impairment was observed in 11.4% (n=18 individuals). Ten out of 18 were diagnosed as CIND and eight with dementia. The prevalence rate of dementia was 4.9% in subjects aged 50 years or over and of CIND was 6.1%. There were no differences in relation to gender or age (five women and five elders) in the CIND group. However, there was a significant difference in relation to schooling, as eight were illiterates. In the demented group (n=8), there was a predominance of females (n=6); all were older than 64 years of age and illiterate.

Considering only the elder group, a prevalence of 12.3% dementia and 7.7% CIND was found. In the adult group, there were no cases of dementia and CIND prevalence was 5.1%. There were no differences in CIND cases for age (µ^2^=0.455, p=0.499) or literacy (µ^2^=0.829, p=0.362). By contrast, analyzing the dementia group, a significant difference was evident for age (µ^2^=13.533, p<0.001) and literacy (µ^2^=4.168, p=0.041).

## DISCUSSION

The aim of the present study was to report the prevalence of CIND and dementia among individuals older than 50 living in communities in the Amazonian Basin. In our sample, the prevalence of cognitive impairment was 11%. Considering only the prevalence of CIND, the rate was 7.7% for elders (>64 years of age). This finding differs from results of other studies. In general, CIND is more frequent than dementia, as individuals can be in a preclinical dementia phase, and have higher risk for conversion. This impairment can be associated with non-degenerative causes, such as vascular, or be secondary to systemic diseases, metabolic disorders or psychiatric conditions, such as depression. Therefore, its frequency is greater and fluctuating during follow-up time.

In Brazil, one study established a CIND rate of 19.5%,^9^ while others have considered the concept of cognitive and functional impairment (including cognitive impairment, dementia and functional decline secondary to any cause).^19^ Chaves et al. have shown a prevalence of mild cognitive impairment of 6.1%, with mean schooling of nine years, superior to the educational level observed in our study.^20^

In the USA, the estimated prevalence of CIND after 70 years of age was 22.2%, ranging from 16% (71-79 years) to 39% (>90 years).^21^ In some reviews this prevalence ranges from 5 to 29%.^22-29^ Among African-Americans, prevalence was 23.4% (increasing with age, from 19.2% to 38%).^29^ In Guadalajara (Mexico), prevalence was 13.8%, with educational level ranging from zero to 23 years. The risk factors associated with cognitive impairment were low educational level, age above 75 years, not married and depression.^30^ A study in Taiwan observed a prevalence of 9.7% with a great influence from schooling;^31^ and in an Italian study conducted in Florence, the incidence of CIND was 21.37/1000 person-years.^22^

Our sample had very low educational level, few vascular risk factors and a lower age relative to other studies. CIND diagnosis in individuals younger than 65 years could involve: low rates due to few systemic diseases; a preclinical stage of dementia at an earlier age associated with low cognitive reserve; and, finally, cognitive impairment due to previous deficits, since childhood. This last factor could be relevant because with the lack of formal education and relatively simple daily activities (or predominantly manual activities) some individuals with borderline intelligence may go unnoticed. Schooling is an important period for the perception of potential deficits. The lower prevalence of CIND and dementia in relation to low educational level in the studied sample might be due to the lower frequency of risk factors for cerebrovascular diseases.

Overall prevalence of dementia in this sample was 4.9% and among the elderly, 12.3%. All of the subjects were illiterate. Considering previous Brazilian studies, these rates are in agreement with the other reports. No studies in subjects 50 years or over are available; our study is unique in this respect. There were no dementia cases among adults (50 to 64 years). The advantage of this study was the rural area and homogenous environment in terms of demands. In Africa, dementia prevalence in adults older than 50 years was estimated to be 2.4%, with higher rates among women and elders (>80 years).^32^ In rural areas of Benin, prevalence of cognitive impairment was 10.4% and of dementia was 2.6% (>64 years); illiteracy was present in 96.6% of the sample.^33^

Meta-analyses have correlated low educational level with a greater risk for dementia.^34,35^ A systematic review pointed out that education is not uniformly described as a risk factor for dementia in all studies. Sixty-one percent of 46 studies had described a significant risk of low education for developing dementia. In nine studies from Latin America, education was linked to dementia risk.^36^

Greater schooling is not a homogeneous influence to protect against dementia, as other factors may also be involved. In our sample, there were no cases in individuals under 64 years; although 67.5% of this group was illiterate. Therefore, cognitive reserve theory cannot explain our observed prevalence. This population is at low risk for vascular diseases, a pathology which can interact with degenerative lesions favoring the development of cognitive impairment.

Limitations of the present study are difficulties diagnosing dementia, mainly in terms of functional evaluation, with no predetermined tool, although comparison within the same environment proved to be the best option. Dementia criteria from DSM-IV do not mention specific methods for evaluating functional impairment. We used the 25^th^ percentile or less than two standard deviations from means as a criterion for cognitive impairment based on MMSE scores, which may have been high. In the study performed by Ganguli et al., the 10^th^ percentile was used.^37^ Besides MMSE scores, we evaluated scores on verbal fluency and the BCSB, tests widely used in Brazil.

In addition, the absolute number of subjects was small, but representative of this population. It is not known whether individuals in a bad state of health had left the reserves for small towns nearby, consequently decreasing dementia rates. However, our findings are in line with other studies.

The main merit of this study is the high homogeneity of the environmental, working, educational, social, and cultural demands. Almost all individuals were born and raised in the region; under the same stimulation throughout life. Another positive aspect is the same access to and quality of education. And finally, participants had the same dietary habits throughout life. Basically, we were able to study the prevalence of cognitive impairment in an almost perfectly controlled sample.
